# Physical Activity and Subsequent Change in Body Weight, Composition and Shape: Effect Modification by Familial Overweight

**DOI:** 10.3389/fendo.2022.787827

**Published:** 2022-02-15

**Authors:** Ina Olmer Specht, Berit Lilienthal Heitmann, Sofus Christian Larsen

**Affiliations:** ^1^ The Parker Institute, Research Unit for Dietary Studies, Bispebjerg and Frederiksberg Hospital, Frederiksberg, Denmark; ^2^ The Boden Group, Faculty of Medicine and Health, Sydney University, Sydney, NSW, Australia; ^3^ The Department of Public Health, Section for General medicine, University of Copenhagen, Copenhagen, Denmark

**Keywords:** physical activity, adiposity, obesity, PAL, genetic

## Abstract

**Background:**

Physical activity (PA) has been shown to attenuate the genetic risk of obesity as measured using polygenic risk scores. However, familial obesity history might be an easier predictor. We examined associations between PA and subsequent changes in BMI, body fat percentage (BF%) and waist circumference (WC) among participants with and without adiposity and a familial overweight.

**Methods:**

In total, 1971 participants from the Danish MONICA cohort were included. Mean differences for 6-year changes in BMI, BF% and WC across PA levels were estimated. Association between walking and biking and subsequent change in adiposity were analysed. Effect modification by familial obesity was assessed by adding product terms to the models.

**Results:**

We observed weak associations between leisure PA level and changes in WC [participants with low PA: 3.4 cm (95%CI: 2.8;4.0), participants with high PA: 2.4 cm (95%CI: 1.8;3.0)], with no evidence of effect modification by familial obesity. We found effect modification in analyses on walking and biking in relation to changes in BMI (P-interaction<0.01) and BF% (P-interaction=0.04), suggesting lower gain with more hours of activity among participants with adiposity and familial overweight.

**Conclusions:**

The results were modest but suggested that PA, especially walking and biking, may prevent future adiposity.

## Introduction

Obesity is one of the leading preventable causes of both morbidity and mortality (1), estimated to account for 8.4% of the total healthcare spending in OECD countries (2). This has raised a serious public health concern, as the prevalence of obesity is increasing on a global level (3). Consequently, knowledge of behavioral risk factors for weight gain should be highly prioritized in order to develop effective public health initiatives targeting the obesity epidemic.

In numerous cross-sectional studies, a sedentary lifestyle has been suggested to play a large role in obesity (4), and evidence also suggest that physical activity has a beneficial effect on regulation of the energy balance, even with some degree of compensation for the higher energy expenditure (5).

Nevertheless, despite reduced PA being a seemingly obvious course of weight gain due to a lower energy expenditure, both observational studies and randomized trials investigating the role of PA in prevention or treatment of obesity have yielded mixed results (4, 6). Some have even suggested obesity as the determinant rather than the consequence of a sedentary lifestyle, which could also explain the strong association between BMI and a sedentary lifestyle observed from cross-sectional studies (7, 8).

Another explanation for the mixed results could be that individuals and/or populations respond differentially to PA in relation to weight gain (9). In support of this, there is a growing body of evidence from studies of specific genetic variants or polygenic risk scores, suggesting that physical activity may modify the genetic susceptibility to develop obesity, with the genetic burden being higher in physically inactive than physically active individuals (10–12). Likewise, a recent genome-wide meta-analysis identified 11 specific genetic variants showing interaction with PA in relation to adiposity.

However, most of these previous studies were based on genetic variants identified in genome-wide association studies (GWAS), and even though hundreds of common variants are associated with obesity, all these variants combined have a poorer predictive ability than traditional hereditary predictors, such as a family history of obesity (13). Despite of this, potential effect modification by familial predisposition to obesity on the association between PA and development of adiposity has only been examined in a few studies (9, 14, 15). These previous family-based cross-sectional or cohort studies, which all investigated twins, generally supported the results from studies using polygenic risk scores, by suggesting that individuals with familial overweight are more prone to increased adiposity if sedentary as compared to individuals without familial predisposition to overweight. However, no longitudinal studies have investigated several measures of adiposity among adult singletons.

Thus, with the present study we examined the longitudinal associations between physical activity and subsequent six-year changes in body mass index (BMI), body fat percentage (BF%) and waist circumference (WC) among participants with and without adiposity and with and without a familial predisposition to overweight.

## Materials and Methods

### Study Population

**Figure 1:**
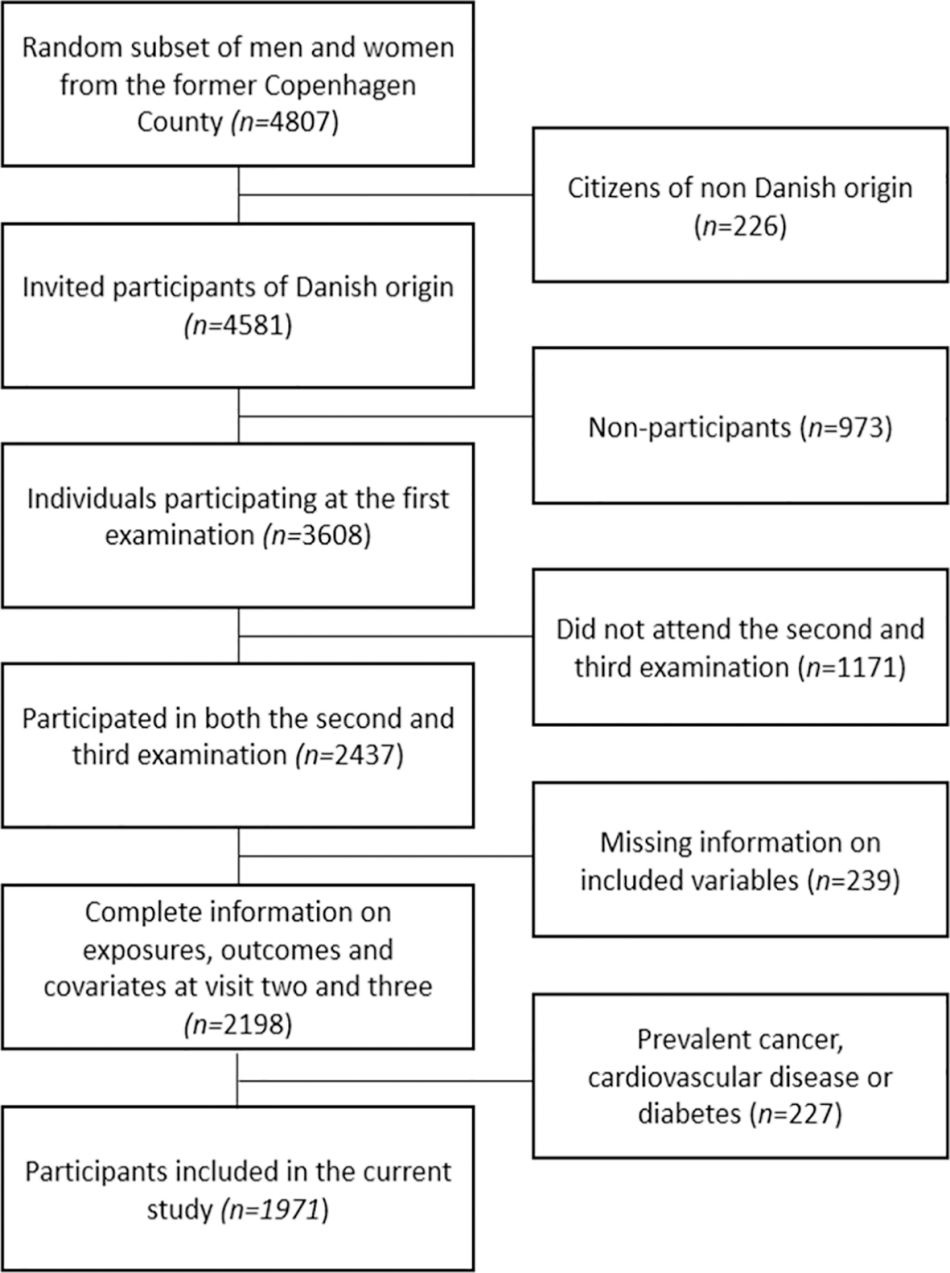
Flowchart showing selection of participants from the MONICA cohort.

The present study was based on data from the Danish MONICA (Monitoring Trends and Determinants in Cardiovascular Disease) cohort. This cohort consists of a random subset of 4,807 men and women born in 1922, 1932, 1942 and 1952, who were selected from residents of 11 surrounding municipalities in the former Copenhagen County (16). Of these, a total of 226 were of non-Danish origin, thus in 1982–83, the 4,581 participants of Danish origin were invited to a health examination, of which 3,608 participated (78.8%) (17). In 1987–88, and again in 1993-94, a second and third invitation was sent to all living participant (17). For the present study we used the second examination as baseline and the third examination as follow-up, as no information on body composition was available from the first examination. Among the 2,437 participants who attended both the second and the third examination, 2,198 had complete information on physical activity level, familial history of obesity, adiposity measures, and covariates. Based on pre-established criteria, we further excluded participants with prevalent cancer (n =53), cardiovascular disease (n = 145) or self-reported diabetes (n = 29), prior to the health examination in 1987–88, ending up with a population of 1,971 men and women. A flowchart illustrating selection of participants from the MONICA cohort can be found in **Figure 1**.

### Ethics

The study was conducted in accordance with the Helsinki Declaration and all participants gave written informed consent. The MONICA study was approved by the Local Ethics Committee of Copenhagen County and the Danish Data Protection Agency.

### Physical Activity

The leisure time physical activity question was based on the single question questionnaire constructed by Saltin and Grimby: “How much do you move and exert yourself physically during leisure time? If your activity varies greatly between, for example summer and winter, try to estimate an average. The question concerns the last year”, with the following categories: inactive (sedentary activities such as reading, watching television and going to the movies), some physical activity (at least 4 hours weekly including for example walking, cycling, construction work, bowling and table tennis), regular hard activity (including for example swimming, tennis and badminton or heavy gardening at least 3 hours weekly) hard activity (elite sports such as swimming, soccer, badminton or long distance running several times a week) (18, 19). Due to few individuals performing hard activity, we combined this category with regular hard activity, and physical activity level was included in the analyses as a categorical variable (low, moderate, high). Furthermore, participants were asked how many minutes they usually spend walking or biking during a day, which was included in the analyses as a continuous variable (hours/day).

### Adiposity Measures

Body weight was measured to the nearest 0.1 kg using a SECA balance scale, with individuals dressed in light clothing or underwear. Height was measured to the nearest 0.5 cm with a rod fixed to a wall and having a sliding horizontal bar (20). Waist circumference (WC) was measured horizontally midway between the lower rib margin and the iliac crest to the nearest 1 cm using a tape measure. Body fat was measured by bioelectric impedance using the BIA-103 body-composition analyzer (RJL Systems, Detroit) following the manufacturer’s instructions. The precision of this method depends on how well the equation is adapted to the population. Thus, an equation was developed on a subset of 139 subjects, from the total population of 2,987 subjects examined in 1987–88, using a four compartment-model based on measurement of body water (dilutometry) and potassium counting (scintigraphy) as the reference:


$$\begin{array}{c} \text{BF (kg)}=14,941+0.819\text{ BW (kg)}-0.279{\text{ height}}^{2}{\text{/R (cm}}^{2}\text{/ohm)} \end{array}$$

In this equation, sex was coded as 1 for men and 0 for women. Fat free mass was calculated by subtracting fat mass from body weight (20).

BMI was calculated by dividing body weight in kg by height in meters squared (kg/m^2^). We used changes in BMI (kg/m^2^), BF% and WC (cm) between baseline (1987–88) and follow-up (1993-94) as the study outcomes.

#### Predisposition to Obesity

The MONICA participants belonging to the highest age and gender specific quartile of BF% were classified as having adiposity and the remaining were classified as without adiposity. Furthermore, all participants were asked if any of their relatives (mother, father, sister, brother or child) ever have had with overweight/obesity. From this information, predisposition was defined in the following four categories: participant without adiposity with no familial history of adiposity (NA/NA), participant without adiposity with family history of adiposity (NA/A), participant with adiposity without family history of adiposity (A/NA) and participant with adiposity and with family history of adiposity (A/A).

### Covariates

Information on several potential confounding factors was collected using questionnaires. Smoking and alcohol consumption were included in the analyses as continuous variables (grams of tobacco/day and units of alcohol/week, respectively). Information on years of regular schooling was categorized as having school education above or below the primary level. Finally, information on age, sex and whether women had entered menopause (yes/no) was also available.

### Statistical Analyses

Analysis of covariance was applied to assess mean differences for changes in BMI, BF% and WC across categories of physical activity (low, moderate, high). First, crude models, including information on change in adiposity measures between baseline and follow-up, baseline physical activity level and baseline measure of outcome, only, were conducted. Secondly, adjusted analyses were carried out, with added information on smoking, alcohol consumption, education, perceived stress, age, sex and menopausal status. The association between walking and biking and subsequent six-year change in adiposity measures were analyzed using multivariate linear regression following the same adjustment scheme as described above. To assess whether associations depended on familial predisposition to overweight, the adjusted analyses were tested for interaction between PA and familial predisposition to overweight by adding product terms to the models. If significant interaction was observed, analyses stratified according to NA/NA, NA/A, A/NA and A/A were presented. Sex interaction was also tested by adding product terms to the models and stratified analyses were conducted if appropriate. Model assumptions (investigating linearity of effects on outcomes, consistency with a normal distribution and variance homogeneity) were assessed for the fully adjusted models through residual plots.

All statistical tests were two-tailed with a significance level at 0.05. Analyses were performed using Stata SE 14 (StataCorp LP, College Station, Texas, USA). Figures were produced using SigmaPlot 13.0 (San Jose, CA, USA).

## Results

As shown in **Table 1**, participants without adiposity (NA) had higher levels of PA and lower levels of adiposity than participants with adiposity (A), regardless of familial overweight status. However, in relation to education the groups differed according to familial overweight status with especially more A/A participants with ≤primary education level (39.6%) as compared to the other participants (27.1% having ≤primary education level among the NA/NA, 29.6% among A/NA, and 30.6% among NA/A, p<0.01).

**Table 1:** Information on physical activity, adiposity measures and covariates^1^.

	NA/NA^2^	NA/A	A/NA	A/A	*P*-value
**n**	1 142	343	294	192	
**Baseline physical activity**					
Activity level (%)					<0.01
Low	20.4	20.1	30.3	31.8	
Moderate	58.3	58.0	56.1	56.8	
High	21.3	21.9	13.6	11.5	
Walking and biking, hours/day	1.1 (1.2)	1.2 (1.3)	1.0 (1.1)	1.1 (1.1)	0.05
**BMI, kg/m^2^**					
Baseline	23.2 (2.4)	23.8 (2.3)	29.2 (3.1)	30.3 (3.4)	<0.01
Follow-up	24.1 (2.7)	24.8 (2.7)	30.0 (3.6)	31.2 (4.1)	<0.01
BF%					
Baseline	24.0 (6.2)	25.8 (6.1)	33.8 (5.9)	36.1 (6.2)	<0.01
Follow-up	26.8 (6.6)	28.4 (6.3)	35.3 (6.3)	37.7 (7.0)	<0.01
**WC, cm**					
Baseline	80.4 (9.3)	81.6 (9.8)	95.1 (11.8)	96.4 (11.9)	<0.01
Follow-up	83.0 (9.9)	85.0 (10.2)	97.4 (11.9)	99.1 (11.9)	<0.01
**Baseline covariates**					
Age, years	48.5 (10.8)	49.0 (10.4)	48.1 (11.0)	49.6 (10.7)	0.35
Sex, % women	49.0	51.6	46.3	54.2	0.31
Height, cm	170.3 (9.1)	170.1 (9.2)	170.3 (9.0)	167.7 (9.5)	0.01
Alcohol intake, units/week	9.0 (11.3)	7.6 (9.8)	11.5 (15.6)	12.3 (38)	0.02
Smoking, g tobacco/day	8.9 (10.4)	7.9 (9.5)	7.8 (11.4)	7.7 (10)	0.04
Smoking, % non-smokers	46.6	49.6	59.9	54.7	<0.01
Education, % ≤primary level	27.1	30.6	29.6	39.6	<0.01
Menopausal status, % post-menopausal among women	45.8	48.6	43.4	52.9	0.45

^1^Results presented as median (SD) unless otherwise stated. ^2^BF%, Bodyfat %; BMI, body mass index; NA, no adiposity MONICA participant; NA, no familial history of adiposity; A, MONICA participant with adiposity; A, familial history of adiposity; and WC, waist circumference.

**Figure 2:**
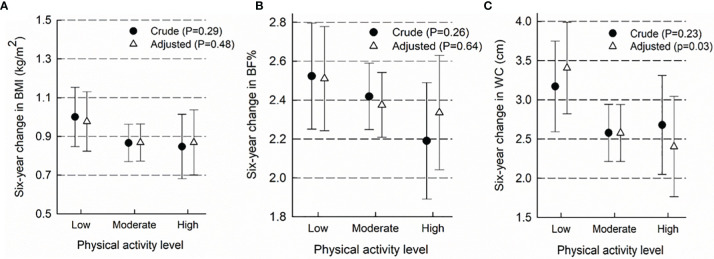
Association between baseline physical activity level and subsequent six-year change in body mass index **(A)**, body fat percentage **(B)** and waist circumference **(C)**. Results presented as mean six-year change in adiposity measures (95% CI) in categories of baseline physical activity level. The crude models include information on exposure, outcome and baseline outcome, only. Adjusted models contain added information on smoking, alcohol consumption, education, sex, menopausal status and age. ^2^BF%, body fat %; BMI, body mass index; and WC, waist circumference.

**Figure 2** shows the association between baseline PA level and subsequent six-year change in BMI (A), BF% (B) and WC (C). Although some trends were seen, particularly for BF%, no significant associations were shown in crude or adjusted models for BMI and BF%. However, an association was observed in the adjusted model of the association between PA level and subsequent six-year change in WC (p=0.03) with the largest gains in WC among participants with low PA. This result was in the same direction as the non-significant results observed for BMI and BF%. We found no evidence of effect modification by familial predisposition to overweight (all p-values for interaction >0.25), and thus stratified analyses are not presented.

**Figure 3:**
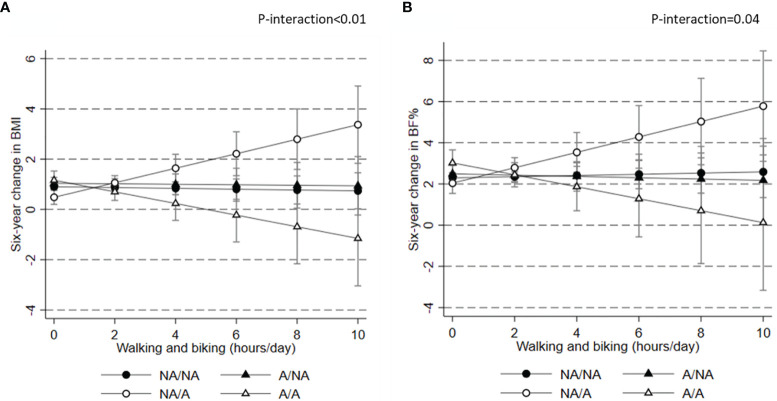
Association between baseline physical activity level and subsequent six-year change in body mass index **(A)** and body fat percentage **(B)** according to predisposition to obesity. ^1^Results presented as mean six-year change in adiposity measures (95% CI) in categories of baseline physical activity level. Results are from adjusted models with information on exposure, outcome, baseline outcome, smoking, alcohol consumption, education, sex, menopausal status and age. ^2^BF%, body fat %; BMI, body mass index; NA, no adiposity MONICA participant; NA, no familial history of adiposity; A, MONICA participant with adiposity; A, familial history of adiposity; and WC, waist circumference.

In the combined sample, we found no evidence of association between daily hours of walking or biking and subsequent six-year change in adiposity (**Table 2**). However, we did find some evidence of effect modification by predisposition to overweight in analyses of change in BMI (P for interaction<0.01) and BF% (P for interaction=0.04), suggesting that irrespective of own level of adiposity there was no relation between walking and biking and weight and adiposity changes for those with no familial overweight. However, among those with familial overweight there was a tendency towards lower weight and fat gain with higher PA among those with own adiposity (A/A), while the opposite was seen among those without own adiposity (NA/A participants) (**Figure 3**).

**Table 2:** Association between walking and biking (hours/day) and subsequent six-year change in adiposity measures^1^.

	Crude	*P*	Adjusted	*P*
Six-year change in BMI^2^, kg/m2	-0.00 (-0.07, 0.06)	0.88	0.01 (-0.05, 0.07)	0.80
Six-year change in BF%	0.01 (-0.10, 0.11)	0.92	0.03 (-0.07, 0.14)	0.52
Six-year change in WC, cm	-0.02 (-0.25, 0.21)	0.87	-0.05 (-0.29, 0.18)	0.64

^1^Results presented as β (95% CI). The crude models include information on exposure, outcome and baseline outcome, only. Adjusted models contain added information on smoking, alcohol consumption, education, sex, menopausal status and age. ^2^BF%, body fat %; BMI, body mass index.

## Discussion

In this relatively large cohort of middle-aged Danish men and women, we found some evidence of a weak inverse association between overall leisure PA level and six year-change in WC. WC is an indicator of abdominal adiposity and associated with cardiovascular disease and Type 2 diabetes, and thus an important measure to affect (21, 22). Although we did observe a statistically significant association suggesting less gain in WC among the most active participants, our results support previous observational studies and randomized trials suggesting that an effect of PA on adiposity in general is modest (4, 7, 23), which could be due to that obesity is a determinant of a sedentary lifestyle (24). Also, no evidence for effect modification by predisposition to overweight was seen. We did however find some indication of effect modification in analyses specifically focusing on walking and biking and six year-changes in BMI or BF%, this depended on both own and on familial overweight. Interestingly, hours spent walking and biking was unrelated with 6-year weight gain among those with no familial overweight both for those with and those without own adiposity. For those with familial overweight, there was lower weight and fat gain with more hours spend walking and biking among those with own adiposity (A/A participants), whereas on the contraire those without adiposity at baseline gained more weight/fat with more hours spend walking and biking (NA/A participants).

Several studies examining gene*PA interactions in relation to BMI have shown that physically active individuals can attenuate their genetic risk of obesity (10, 11, 25). While we were unable to directly confirm this in our study, we did find the lowest weight and fat gains with more hours spend walking and biking among A/A participants, which were likely also the most genetically predisposed of the participants. The reason our results are not completely consistent with previous studies based on genetic variants can be due to many factors. We did not have information on genetic variants but used familial overweight in combination with the participants own adiposity status as a proxy for a genetic predisposition. This obviously introduces a limitation, as the underlying cause of obesity could be multifactorial and not only related to genetics. Likewise, the family predisposition to overweight was self-reported and only information on at least one relative was given. Individuals with two overweight relatives will likely have a higher genetic or environmental predisposition to obesity. Few studies in this area have focused directly on familial history of overweight. However, a previous study among Finnish twins also showed somewhat similar results to ours and the existent genetically based evidence, suggesting that a sedentary lifestyle mainly have an obesity-promoting effect in those with a higher genetic predisposition (15).

Our study has several strengths, including a relatively large sample size with validated information on PA and longitudinal information on adiposity measures, including body composition, as well as questionnaire data on several lifestyle factors, allowing us to adjust for potential confounding. The leisure time physical activity questionnaire constructed by Saltin and Grimby has been proven to categorize individuals reliable into PA level groups of four (18). The questionnaire comprises a comprehensive branch of PA, such as sports, transport, homework and gardening, however the questionnaire consists of a single question where the participant rates her or him-self and does not include occupational activity, nor information on duration, frequency or intensity of the activity. The information on PA was self-reported which might cause response bias (26), potentially causing more participants to report a higher PA level. However, since our sample size was large, this error might be less relevant. An objective measure of PA activity like accelerometer measurements would have been preferred, but the used questionnaire has previously shown good reliability and validity (27). To gain additional knowledge of the participants PA, we included information on hours per day walking and biking. We had three measures of adiposity, BMI, BF% and WC. BMI, which is an often used measure of adiposity, may not be the best measure of obesity in relation to PA since it does not differentiate between lean mass and fat (22). We had information on BF% and WC which both are good indicators of obesity. BF% is a measure of the relative contribution of fat to the total body mass comprised of lean and fat, and WC relates to abdominal adiposity. Both measures are good predictor of cardiovascular disease risk factors, type 2 diabetes, and metabolic syndrome (21, 22, 28). Finally, the MONICA study had a very high participation rate of 78.8%, and the participants included have previously been shown to be reasonable representative of the general Danish population, though with a slightly underrepresentation of people employed in agriculture, horticulture and fishery as well as self-employed and unskilled workers (29).

However, our study also has some limitations. As in other cohort studies, we cannot exclude that unmeasured or residual confounding have affected our results. Familial predisposition to overweight was self-reported and might, like the PA questionnaire, have been prone to response bias which could bias the observed interactions in any direction depending on the participants own obesity status. The number of participants with low PA was higher among the participants with adiposity, a reason could be that obesity is a determinant for low PA rather than low PA is a determinant for obesity (7, 8). In our study, the participants with obesity had the lowest SES, which also has been shown in other studies (30). Low SES has besides obesity been linked to physical inactivity, unhealthy dietary patterns and stress (31, 32), all factors that can be related to obesity. Moreover, we did not have information available on dietary intake for all the included participants, and thus we were unable to consider both ends of the energy-balance equation, which is probably our main limitation since the effect of PA on weight is highly dependent of energy intake. However, given the high degree of measurement error related common dietary assessment tools, we find it unlikely that including this information would have changed our results substantially. Although we analyzed a relatively large cohort, our sample size may also have been too small to detect minor interaction effects. Consequently, it is possible that we over-looked some associations because of a lack of statistical power.

In conclusion, an effect of total leisure time PA on long-term adiposity was modest among the middle-aged Danish men and women in this study, however we did find some evidence suggesting that PA may prevent gain in adiposity, specifically in the abdominal region, but not related to familial predisposition to obesity. Finally, our results suggest that walking and biking may be a more beneficial weight management tool for individuals with adiposity and a family history to overweight. Due to the design of our study, we cannot confirm with certainty whether this interaction reflects genetics or environment.

## Data Availability Statement

The raw data supporting the conclusions of this article will be made available by the authors, without undue reservation.

## Ethics Statement

The studies involving human participants were reviewed and approved by The Local Ethics Committee of Copenhagen County. The patients/participants provided their written informed consent to participate in this study.

## Author Contributions

SL and BH designed the study. SL analyzed the data, IOS drafted the manuscript. All authors approved the final manuscript for submission.

## Funding

The Parker Institute is funded by a core grand from the OAK Foundation (OCAY-13-309). The funder had no role in the design of the study and did not participate in the data collection, analysis and interpretation. In addition, the funder did not participate in the preparation of the manuscript or decision to publish.

## Conflict of Interest

The authors declare that the research was conducted in the absence of any commercial or financial relationships that could be construed as a potential conflict of interest.

## Publisher’s Note

All claims expressed in this article are solely those of the authors and do not necessarily represent those of their affiliated organizations, or those of the publisher, the editors and the reviewers. Any product that may be evaluated in this article, or claim that may be made by its manufacturer, is not guaranteed or endorsed by the publisher.
